# Supplementary material for: Exposure to conflicts and the continuum of maternal healthcare: Analyses of pooled cross-sectional data for 452,192 women across 49 countries and 82 surveys
Source: PLoS Med. 2021 Sep 28;18(9):e1003690. doi: 10.1371/journal.pmed.1003690 (PMC8478181; doi:10.1371/journal.pmed.1003690)
Supplement: S1 Table — STROBE, Strengthening the Reporting of Observational Studies in Epidemiology. (DOCX) [file pmed.1003690.s001.docx]

**S1Table. STROBE Checklist**

|  | **Item No** | **Recommendation** | **Section and paragraph** |
| --- | --- | --- | --- |
| **Title and abstract** | 1 | (*a*) Indicate the study’s design with a commonly used term in the title or the abstract | Title and Abstract |
|  |  | (*b*) Provide in the abstract an informative and balanced summary of what was done and what was found | Abstract |
| **Introduction** | | |  |
| Background/rationale | 2 | Explain the scientific background and rationale for the investigation being reported | Introduction, paragraph 1, 2 and 3 plus Figure 1 |
| Objectives | 3 | State specific objectives, including any prespecified hypotheses | Introduction, paragraph 4 |
| **Methods** | | |  |
| Study design | 4 | Present key elements of study design early in the paper | Methods, section Data sources paragraph 1 |
| Setting | 5 | Describe the setting, locations, and relevant dates, including periods of recruitment, exposure, follow-up, and data collection | Methods, section Data sources paragraph 2 and 3 |
| Participants | 6 | (*a*) Give the eligibility criteria, and the sources and methods of selection of participants. Describe methods of follow-up | Methods, section Data sources paragraph 4 and 5, section Study population paragraph 1 plus Figure 2 |
|  |  | (*b*) For matched studies, give matching criteria and number of exposed and unexposed | Methods, section Data sources paragraph 3 |
| Variables | 7 | Clearly define all outcomes, exposures, predictors, potential confounders, and effect modifiers. Give diagnostic criteria, if applicable | Methods, section Outcome variables paragraph 1, 2 plus Figure 3, section Explanatory variables, paragraph 1 – 6 plus Table 1 and 2 |
| Data sources/ measurement | 8* | For each variable of interest, give sources of data and details of methods of assessment (measurement). Describe comparability of assessment methods if there is more than one group | Methods, section Data sources paragraph 1, section Outcome variables paragraph 1, section Explanatory variables paragraph 1 – 6 |
| Bias | 9 | Describe any efforts to address potential sources of bias | Methods, section Explanatory paragraph 5, section Empirical strategy paragraph 2 |
| Study size | 10 | Explain how the study size was arrived at | Methods, section Study population paragraph 1 |
| Quantitative variables | 11 | Explain how quantitative variables were handled in the analyses. If applicable, describe which groupings were chosen and why | Methods, section Outcome variables paragraph 1, section Explanatory variables paragraph 3, 4, 5 and 6 |
| Statistical methods | 12 | (*a*) Describe all statistical methods, including those used to control for confounding | Methods, section Empirical strategy paragraph 1 and 2 |
|  |  | (*b*) Describe any methods used to examine subgroups and interactions | NA |
|  |  | (*c*) Explain how missing data were addressed | NA |
|  |  | (*d*) If applicable, explain how loss to follow-up was addressed | NA |
|  |  | (*e*) Describe any sensitivity analyses | NA |
| **Results** | | |  |
| Participants | 13* | (a) Report numbers of individuals at each stage of study—e.g. numbers potentially eligible, examined for eligibility, confirmed eligible, included in the study, completing follow-up, and analysed | Methods, section Study population paragraph 1 |
|  |  | (b) Give reasons for non-participation at each stage | Methods, section Study population paragraph 1 |
|  |  | (c) Consider use of a flow diagram | Methods, section Study population Figure 1 |
| Descriptive data | 14* | (a) Give characteristics of study participants (eg demographic, clinical, social) and information on exposures and potential confounders | Methods, section Explanatory variables paragraph Table 2,  Results, section Summary statistics paragraph 1 – 4 plus Figure 4 |
|  |  | (b) Indicate number of participants with missing data for each variable of interest | Methods, section Study population paragraph Figure 2 |
|  |  | (c) Summarise follow-up time (e.g., average and total amount) | Results, section Summary statistics paragraph 1 |
| Outcome data | 15* | Report numbers of outcome events or summary measures over time | Results, section Summary statistics paragraph 1 |
| Main results | 16 | (*a*) Give unadjusted estimates and, if applicable, confounder-adjusted estimates and their precision (e.g., 95% confidence interval). Make clear which confounders were adjusted for and why they were included | Table 2, 3, 4, 5 and 6 |
|  |  | (*b*) Report category boundaries when continuous variables were categorized | Methods, section Explanatory variables paragraph 4 and 6 |
|  |  | (*c*) If relevant, consider translating estimates of relative risk into absolute risk for a meaningful time period | NA |
| Other analyses | 17 | Report other analyses done—e.g. analyses of subgroups and interactions, and sensitivity analyses | Results Table 4 |
| **Discussion** | | |  |
| Key results | 18 | Summarise key results with reference to study objectives | Discussion, paragraph 1, 2 and 3 |
| Limitations | 19 | Discuss limitations of the study, taking into account sources of potential bias or imprecision. Discuss both direction and magnitude of any potential bias | Discussion, section Strength and limitations paragraph 1,2 and 3 |
| Interpretation | 20 | Give a cautious overall interpretation of results considering objectives, limitations, multiplicity of analyses, results from similar studies, and other relevant evidence | Discussion, section Strengths and limitations paragraph 2 |
| Generalisability | 21 | Discuss the generalisability (external validity) of the study results | Discussion, section Strength and limitations paragraph 3 |
| **Other information** | | |  |
| Funding | 22 | Give the source of funding and the role of the funders for the present study and, if applicable, for the original study on which the present article is based | NA |

*Give information separately for exposed and unexposed groups.

**Note:** An Explanation and Elaboration article discusses each checklist item and gives methodological background and published examples of transparent reporting. The STROBE checklist is best used in conjunction with this article (freely available on the Web sites of PLoS Medicine at http://www.plosmedicine.org/, Annals of Internal Medicine at http://www.annals.org/, and Epidemiology at http://www.epidem.com/). Information on the STROBE Initiative is available at http://www.strobe-statement.org.
