# Supplementary material for: Exposure to conflicts and the continuum of maternal healthcare: Analyses of pooled cross-sectional data for 452,192 women across 49 countries and 82 surveys
Source: PLoS Med. 2021 Sep 28;18(9):e1003690. doi: 10.1371/journal.pmed.1003690 (PMC8478181; doi:10.1371/journal.pmed.1003690)
Supplement: S2 Table — (DOCX) [file pmed.1003690.s002.docx]

**Table S2. Number of observations by information availability**

| No. | Country | Total sample | Sample with ANC information | Sample with information on delivery | Sample with information on PNC | Final data  with information on all our variables of interest |
| --- | --- | --- | --- | --- | --- | --- |
| 1 | Afghanistan | 32,712 | 19,353 | 32,204 | 19,687 | 18,984 |
| 2 | Albania | 4,378 | 3,651 | 4,352 | 1,595 | 1,306 |
| 3 | Angola | 14,322 | 8,839 | 14,081 | 0 | 0 |
| 4 | Armenia | 4,627 | 3,615 | 4,598 | 2,491 | 2,160 |
| 5 | Azerbaijan | 2,297 | 1,642 | 2,280 | 0 | 0 |
| 6 | Bangladesh | 29,697 | 22,089 | 26,474 | 22,621 | 21,319 |
| 7 | Bolivia | 19,053 | 13,672 | 18,888 | 12,624 | 8,128 |
| 8 | Burkina | 25,689 | 17,623 | 25,589 | 16,703 | 14,275 |
| 9 | Burundi | 20,934 | 13,557 | 20,069 | 4,908 | 4,524 |
| 10 | Cambodia | 23,687 | 18,406 | 23,582 | 22,333 | 18,261 |
| 11 | Cameroon | 19,857 | 12,822 | 19,603 | 10,855 | 8,656 |
| 12 | Chad | 24,258 | 14,390 | 24,067 | 16,638 | 14,030 |
| 13 | Colombia | 44,136 | 35,660 | 36,026 | 32,250 | 0 |
| 14 | Comoros | 3,149 | 1,730 | 3,082 | 2,005 | 1,707 |
| 15 | Congo | 14,164 | 9,925 | 14,014 | 6,458 | 5,752 |
| 16 | Congo Democratic | 27,708 | 16,505 | 27,242 | 20,233 | 15,418 |
| 17 | Egypt | 40,571 | 40,221 | 40,536 | 19,866 | 19,667 |
| 18 | Eswatini | 2,812 | 2,072 | 2,795 | 2,788 | 1,249 |
| 19 | Ethiopia | 22,295 | 14,911 | 22,030 | 7,740 | 7,565 |
| 20 | Gambia | 8,088 | 5,377 | 8,029 | 5,370 | 5,146 |
| 21 | Ghana | 12,720 | 9,023 | 12,631 | 6,517 | 5,357 |
| 22 | Guatemala | 12,440 | 9,537 | 12,377 | 9,538 | 8,979 |
| 23 | Guinea | 21,354 | 14,587 | 21,241 | 9,375 | 7,533 |
| 24 | Guyana | 2,178 | 1,331 | 2,117 | 2,110 | 1,140 |
| 25 | Haiti | 19,792 | 14,610 | 19,562 | 11,393 | 9,084 |
| 26 | Honduras | 21,688 | 16,769 | 21,583 | 8,713 | 8,140 |
| 27 | Indonesia | 54,514 | 45,553 | 54,052 | 15,152 | 14,951 |
| 28 | Jordan | 41,094 | 20,777 | 31,342 | 17,108 | 13,389 |
| 29 | Kenya | 32,992 | 22,784 | 32,598 | 10,634 | 8,520 |
| 30 | Kyrgyz | 4,363 | 3,067 | 4,335 | 3,137 | 3,045 |
| 31 | Lesotho | 10,834 | 8,499 | 10,673 | 9,419 | 7,307 |
| 32 | Liberia | 13,405 | 8,652 | 13,193 | 11,062 | 7,148 |
| 33 | Madagascar | 17,863 | 12,025 | 17,675 | 3,308 | 1,979 |
| 34 | Mali | 34,504 | 21,770 | 34,181 | 6,723 | 6,366 |
| 35 | Moldova | 1,552 | 1,311 | 1,548 | 1,537 | 1,285 |
| 36 | Morocco | 6,180 | 4,742 | 6,136 | 6,159 | 4,721 |
| 37 | Mozambique | 21,428 | 14,418 | 21,005 | 12,380 | 9,640 |
| 38 | Myanmar | 4,815 | 3,833 | 4,802 | 0 | 0 |
| 39 | Namibia | 10,214 | 6,760 | 10,137 | 3,961 | 1,686 |
| 40 | Nepal | 16,127 | 12,266 | 15,897 | 4,079 | 4,012 |
| 41 | Niger | 21,751 | 13,511 | 21,591 | 16,772 | 13,309 |
| 42 | Nigeria | 100,082 | 61,457 | 98,463 | 52,111 | 36,703 |
| 43 | Pakistan | 33,648 | 21,334 | 33,497 | 16,551 | 7,398 |
| 44 | Papua New Guinea | 9,514 | 6,268 | 8,967 | 0 | 0 |
| 45 | Peru | 72,714 | 59,295 | 65,379 | 0 | 0 |
| 46 | Philippines | 31,484 | 22,850 | 31,139 | 18,931 | 14,422 |
| 47 | Rwanda | 30,996 | 21,168 | 30,301 | 18,325 | 14,449 |
| 48 | Senegal | 69,681 | 45,620 | 69,198 | 35,224 | 31,714 |
| 49 | Sierra | 17,569 | 10,816 | 17,247 | 8,458 | 6,572 |
| 50 | South Africa | 3,548 | 2,942 | 3,532 | 3,462 | 3,421 |
| 51 | Tajikistan | 11,208 | 7,663 | 11,146 | 0 | 0 |
| 52 | Tanzania | 18,256 | 12,344 | 17,988 | 0 | 0 |
| 53 | Togo | 6,979 | 5,001 | 6,938 | 5,002 | 4,773 |
| 54 | Turkey | 12,038 | 10,301 | 11,967 | 2,039 | 0 |
| 55 | Uganda | 31,769 | 19,989 | 31,424 | 4,887 | 4,537 |
| 56 | Ukraine | 1,221 | 926 | 1,214 | 0 | 0 |
| 57 | Yemen | 16,093 | 10,467 | 15,891 | 10,424 | 10,314 |
| 58 | Zambia | 29,817 | 20,661 | 29,498 | 9,337 | 8,113 |
| 59 | Zimbabwe | 16,941 | 13,182 | 16,633 | 4,395 | 4,038 |
|  | Total | 1,279,800 | 894,169 | 1,238,639 | 585,388 | 452,192 |
