# Supplementary material for: Exposure to conflicts and the continuum of maternal healthcare: Analyses of pooled cross-sectional data for 452,192 women across 49 countries and 82 surveys
Source: PLoS Med. 2021 Sep 28;18(9):e1003690. doi: 10.1371/journal.pmed.1003690 (PMC8478181; doi:10.1371/journal.pmed.1003690)
Supplement: S3 Table — (DOCX) [file pmed.1003690.s003.docx]

**Table S3. Conflict status during 1997 to 2018 in 49 countries in the final sample**

| No. | Country | No. of all conflicts | Total number of deaths | Start year of the earliest conflict | End year of the latest conflict |
| --- | --- | --- | --- | --- | --- |
| 1 | Afghanistan | 27,787 | 190,279 | 1997 | 2018 |
| 2 | Albania | 4 | 6 | 1999 | 1999 |
| 3 | Armenia | 19 | 34 | 2005 | 2018 |
| 4 | Bangladesh | 429 | 720 | 1997 | 2018 |
| 5 | Bolivia | 10 | 52 | 1998 | 2000 |
| 6 | Burkina Faso | 68 | 206 | 2016 | 2018 |
| 7 | Burundi | 1,296 | 12,126 | 1997 | 2018 |
| 8 | Cambodia | 99 | 740 | 1997 | 2012 |
| 9 | Cameroon | 599 | 4,254 | 1998 | 2018 |
| 10 | Chad | 245 | 6,923 | 1997 | 2018 |
| 11 | Comoros | 5 | 96 | 1997 | 1998 |
| 12 | Congo | 211 | 15,525 | 1997 | 2018 |
| 13 | DR Congo | 3,515 | 73,275 | 1997 | 2018 |
| 14 | Egypt | 454 | 3,849 | 1997 | 2018 |
| 15 | Eswatini | 0 | 0 |  |  |
| 16 | Ethiopia | 1,714 | 87,882 | 1997 | 2018 |
| 17 | Gambia | 2 | 8 | 2005 | 2005 |
| 18 | Ghana | 16 | 180 | 2000 | 2010 |
| 19 | Guatemala | 25 | 163 | 1997 | 2015 |
| 20 | Guinea | 78 | 1,073 | 1998 | 2018 |
| 21 | Guyana | 3 | 29 | 2008 | 2008 |
| 22 | Haiti | 32 | 304 | 1999 | 2018 |
| 23 | Honduras | 32 | 236 | 1999 | 2016 |
| 24 | Indonesia | 1,567 | 7,349 | 1997 | 2018 |
| 25 | Jordan | 5 | 93 | 2005 | 2016 |
| 26 | Kenya | 840 | 4,851 | 1997 | 2018 |
| 27 | Kyrgyzstan | 47 | 238 | 1999 | 2010 |
| 28 | Lesotho | 4 | 68 | 1998 | 1998 |
| 29 | Liberia | 136 | 6,218 | 1997 | 2003 |
| 30 | Madagascar | 43 | 208 | 2002 | 2017 |
| 31 | Mali | 637 | 4,238 | 1997 | 2018 |
| 32 | Moldova | 0 | 0 |  |  |
| 33 | Morocco | 2 | 34 | 2003 | 2003 |
| 34 | Mozambique | 77 | 272 | 2004 | 2018 |
| 35 | Namibia | 18 | 72 | 1999 | 2002 |
| 36 | Nepal | 5,585 | 12,259 | 1997 | 2009 |
| 37 | Niger | 155 | 1,684 | 1997 | 2018 |
| 38 | Nigeria | 4,026 | 51,798 | 1997 | 2018 |
| 39 | Pakistan | 5,595 | 40,568 | 1997 | 2018 |
| 40 | Philippines | 3,046 | 14,107 | 1997 | 2018 |
| 41 | Rwanda | 204 | 8,591 | 1997 | 2018 |
| 42 | Senegal | 186 | 1,220 | 1997 | 2018 |
| 43 | Sierra Leone | 775 | 11,811 | 1997 | 2002 |
| 44 | Tajikistan | 75 | 906 | 1997 | 2018 |
| 45 | Togo | 97 | 445 | 1998 | 2005 |
| 46 | Uganda | 1,427 | 11,607 | 1997 | 2018 |
| 47 | Yemen | 2,225 | 24,767 | 2003 | 2018 |
| 48 | Zambia | 3 | 8 | 2000 | 2001 |
| 49 | Zimbabwe | 51 | 261 | 1998 | 2018 |
|  | Total | 63,469 | 601,633 |  |  |
