# Supplementary material for: Exposure to conflicts and the continuum of maternal healthcare: Analyses of pooled cross-sectional data for 452,192 women across 49 countries and 82 surveys
Source: PLoS Med. 2021 Sep 28;18(9):e1003690. doi: 10.1371/journal.pmed.1003690 (PMC8478181; doi:10.1371/journal.pmed.1003690)
Supplement: Attachment — Submitted filename: Point by Point Response to Editors__22.4.21_.docx [file pmed.1003690.s004.docx]

**Point-by-point response to Editor and Reviewers’ comments**

Dear Editor

We are grateful to you and the reviewers for their comments and suggestions. The paper has greatly improved from the incorporation of these suggestions. Below we describe the manner in which we have addressed each of the suggestions made by the Editor and Reviewers. Our responses appear in italics.

**Response to Editor’s comments**

Comment 1: Please adapt the title to include a study descriptor after the colon, e.g., "...: A cohort study".

*Authors’ response: Thank you for this suggestion. We have now made this change.*

Comment 2: Please add a new final sentence to the "Methods and findings" subsection of your abstract, beginning "Study limitations include ..." and quoting 2-3 of the study's main limitations.

*Authors’ response: Thank you. We have now added some Study limitations (pp. 1-2).*

Comment 3: Given the study design, please avoid language implying causality, e.g., "effect" late in your abstract.

*Authors’ response: We have addressed this comment and removed any language implying causality.*

Comment 4: Please use the active voice in 1-2 points in your Author Summary (e.g., "We combined ...").

*Authors’ response: The suggestion is well taken. We have now addressed this comment.*

Comment 5: Please trim the final paragraph of the Introduction substantially. A brief description of the study's aim will suffice here, and discussion of the methods used and their merits can be moved to the Discussion section.

*Authors’ response: Thanks for this comment. This suggestion has now been incorporated.*

Comment 6: Early in the Methods section, please state whether or not the study had a protocol or prospective analysis plan, and if so attach the relevant document(s) as a supplementary file(s), referred to in the text. Please highlight analyses that were not prespecified.

*Authors’ response: Our study design does not have a protocol. We have attached a Supporting Information file including a STROBE checklist.*

Comment 7: Please avoid claims of "the first" and the like, e.g., early in your Discussion section. Where necessary, please add "to our knowledge" or similar.

*Authors’ response: We have rephrased this sentence as suggested by the Editors (p. 25).*

Comment 8: Throughout the paper, please remove spaces from the reference call-outs (e.g., "... care usage [4,11,12], ").

*Authors’ response: The suggestion is incorporated in the revised manuscript.*

Comment 9: Please review your reference list to ensure that references are formatted consistently and according to journal style. No more than 6 author names should be listed, followed by "et al.". Italics should be converted to plain text. Please abbreviate journal names consistently (e.g., "Lancet").

*Authors’ response: The suggestion is incorporated in the revised manuscript.*

Comment 10: Please add a completed checklist for the most appropriate reporting checklist, e.g., RECORD, as a supplementary file, labelled "S1_RECORD_Checklist" or similar and referred to as such in your Methods section. In the checklist, please refer to individual items by section (e.g., "Methods") and paragraph number rather than by line or page numbers, as the latter generally change in the event of publication.

*Authors’ response: The suggestion has been incorporated in the revised manuscript (p. 29).*

**Response to Reviewer #1:**

Comment 1: I confine my remarks to statistical aspects of this paper. Unfortunately, there were some fairly serious issues that need to be addressed.

One is how the fact that the data are nested was dealt with. There are a variety of approaches such as GEE, MLM and robust standard errors. I think a nonlinear MLM (multilevel model) is indicated here, but the other methods would also be OK.

*Authors’ response: While our data has multi-level structure, our primary interest is in a single-level relationship rather than in the parameter heterogeneity across different levels of analysis. Thus we do not employ multi-level modelling approach in the current analysis, but we do address the clustering of standard errors in our multilevel data structure by estimating cluster-robust standard errors that adjust the standard errors for correlation within provinces. We have noted this in the revised paper (p. 15-16).*

Comment 2: Another is that the equation shown on p. 9 is not logistic regression, it is linear regression. You need to either change the DV by taking the logit or else exponiiante the right side.

*Authors’ response: The originally submitted version of the model presented the latent variable model. In the revised version of the paper we have drawn on it to explicitly present the binary logistic regression model estimated (equation (2) on p.15).*

Comment 3: Finally, causal language should be avoided; even with controls, this is an observational study.

*Authors’ response: Thank you. We have addressed this comment in the revised manuscript.*

Comment 4: Page 8  Don't make terciles or otherwise categorize continuous variables. This increases both type 1 and type 2 error and introduces "magical thinking" - that is, that something special happens at the cut-offs’.

*Authors’ response: Our measure of intensity of conflict is defined as the average number of deaths per year of conflict exposure and is skewed towards 0, with 62% of women in the sample not exposed to any violent events that resulted in positive deaths. Thus, we think that it is unlikely that the relationship between this measure and our outcome measures is linear.*

*With this consideration in mind, as a first step, we exploit categorical measures of conflict intensity. Next, as a second step, we address this comment more explicitly by employing the square root of the number of deaths (in thousands) per year of conflict exposure as a robustness check. This approach to defining the conflict exposure intensity measure follows existing studies and mitigates the common concerns around categorizing continuous variables. Both approaches yield qualitatively similar results. Please see new table 6 on p. 24 and the discussion on p. 13.*

Comment 5: Talking about the "mean" of a dichotomous variable (like each stage of CoC) is a bit odd. Usually people would say "proportion" - but this works out the same.

*Authors’ response: We have addressed this issue in the revised version of the paper.*

Comment 6: The figures seem to have errors. By design, each stage of CoC has to be less common than earlier ones. But this is not reflected in the figure. E.g. for Afghanistan, the mean for level 3 is higher than for level 2. For Albania, they seem to be equal. Problems like this occur for other countries, too.  So, either something is wrong in what you plotted, or I am confused (but if I am confused, others are likely to be confused as well).

*Authors’ response: Thanks for pointing this out. In the original version of the paper, we had accidentally made the Y Axis scale uniform for the figures across all components of CoC, so that they are compared well on a similar scale. This error has now been amended in the revised figures. The scale now varies from “0” to “1” for all the figures.*

**Response to Reviewer #2:**

**Comment 1:** The article "Exposure to conflicts and its influence on continuum of maternal health care: Analyses of 452,192 women across 49 countries and 82 surveys" by Rammohan explores the change in the odds of receiving any of a set of maternal care components in relation to exposure to conflict. The article is original, well-conceived, well-written, and compelling.
The only substantive comment of note is that the exposure is defined at the provincial level. Some provinces (by which I assume the authors mean the first administrative level - the terminology differs by country) are small (and therefore exposure is well-measured), while others are large, and exposure may be measured with noise. Along a similar vein, using the province boundary may miss nearby conflicts just on the other side of the province boundary. An improvement would be to allow linkage by lat-long coordinates. However, that will likely only improve precision (classical measurement error in the exposure variables), and is not critical for inference in this paper.

*Authors’ response: We are grateful to Reviewer 2 for their positive feedback on our paper. We agree that the inclusion of information on lat-long coordinates would improve precision. However, we have chosen not to do so for the following reasons. Firstly, the GPS data is available for a smaller sample of DHS countries. We chose not to compromise on the number of countries represented in the sample given the focus of the current study on providing a large multi-country evidence on the link between conflict exposure and its influence on continuum of maternal health care. Secondly, to safeguard the respondent confidentiality, DHS randomly displays the GPS latitude/longitude positions up to 2 kilometres in urban areas and 5 kilometres in rural areas. As a result, we are not able to accurately capture the location of residence for matching purposes. We have now included a para on p. 7 of the amended version justifying this decision.*

**Comment 2:** The main non-substantive comments is that the manuscript is written as if geared for economists, and towing closer to medical literature templates may make it more impactful among medical/health readership. Suggestions may be to shorten/tighten the intro; remove boldface parts; simplify some of the methods; extend discussion and include some limitations.

*Author’s response: Thank you for this feedback. We have addressed this issue in the revised version of the paper. We have now made these changes. Please see new Discussion on pages 25-28, new methods sections (on pages 5-16) and a cut back Introduction (pages 3-5).*

Comment 3: The article is clear, lucid, and a pleasure to read. Overall this makes a really nice and important contribution to the thickening literature about the role of conflict in eroding the health and well-being of women and children.

*Authors’ response: Thanks for this comment.*

**Response to Reviewer #3:**

**Comment 1:** Background: This section is rather thin and lacks the depth of the rationale for the study. It is not a surprise that conflict affects access to services. There is a lot of literature on this and it would have been useful to include even if small framework in what way the conflict affects this. For example evidence on increase of c-sections and delay of immunizations are key to understand the interaction between conflict and maternal health outcomes.

*Authors’ response: Thanks for pointing this out. It appears that we did not articulate the rationale for our study clearly in the previous version of the paper. We have made our contributions clearer in the revised version of the paper. As pointed out in this revised paper, while the link between maternal health outcomes and conflict has been extensively studied, the focus of our study is on the association between conflict and Continuum of care. This is a concept that our knowledge has only been studied in single country or regional focused studies.*

*With regards to a framework, we have now added a Pathway diagram (Pls see Figure 1), where we show the potential pathways between conflict and CoC.*

**Comment 2:** Another consideration missing is type of place of conflict and how differently this might affect the access.

*Authors’ response: Thanks for pointing this out. We have addressed this issue in the revised version of the paper. We have conducted new analyses to assess heterogeneous effects of conflict by sub-sample of difference place of residence: Rural and Urban areas. Please see the results presented in Table 4 and the associated discussion on pp. 25-26.*

**Comment 3:** Recent papers on Palestine (Leone et al and Siam et al.) have highlighted this mainly given different intensities (tackled by this paper) in different parts of the country. Basically the paper in the background and the conclusions needs to answer the so what question beyond the data contribution.

*Authors’ response: Thanks for pointing us to these papers- we have now included them in our text and reference list. As we have acknowledged in our original draft that there are studies at the individual country level, whereas our paper considers pooled cross-sectional data of 452,192 women across 49 countries and 82 surveys for which consistent data are available. Moreover, our focus on CoC is unique to the best of our knowledge. Moreover, single country papers are context specific. Our paper uses robust quantitative evidence based on relatively a large sample to show that the links between conflict and CoC is robust across time and countries.*

**Comment 4:** Methods: The key contribution of this paper is the inclusion of a large number of datasets and most importantly the inclusion of information of the Upsala datasets.
There are a few points that need clarification such as how were the data pooled together?

*Authors’ response: Thanks for pointing this out. We have addressed this issue in the revised version of the paper. Figure 2 explains the process through which the population sample was pooled using the two datasets. The methodology section has now been substantially revised and includes the details of the data pooling process (pp. 6-7).*

**Comment 5: Were synthetic cohorts used?**

*Authors’ response: No, we did not use synthetic cohorts.*

**Comment 6:** Can you include more information on how the exposure was considered? Were past births included? Did you reconstruct the birth histories? How was the file constructed?

*Authors’ response: Our variable of interest is the Continuum of care. Therefore we needed information on four components beginning with antenatal care. We considered only the last birth in the five years prior to the interview date since information on the components of CoC are only available for the last birth. We have now clarified the construction of the exposure in the Methods section. Please see page 6-7.*

**Comment 7: There needs to be a rationale for the outcomes. Eg: why 4 visits-not obvious to everyone.**

*Authors’ response: The rationale for ‘At least 4 or more ANCs’ is well-established as the WHO’s recommendation, and accordingly all DHS countries use it as one of the key maternal health care indicators (https://www.who.int/data/gho/indicator-metadata-registry/imr-details/80).*

**Comment 8: There needs to be a discussion on the quality of the conflict data, in particular how it varies across countries. This needs to go in the limitations.**

*Authors’ response: The UCDP uses a consistent measure of conflict for all countries, and is generally considered to be reliable. However, we acknowledge that there is potential for measurement errors in large cohort multi-country studies. We have therefore included this point among the limitations of our study.*

**Comment 9: Why was education not included?**

*Authors’ response: Education was included in the analysis of original draft submitted to the journal. We have now highlighted it specifically in the text.*

**Comment 10: Was wealth calculated separately for urban and rural areas to account for different weight? If not it needs to be acknowledged in the limitations.**

*Authors’ response: Yes, the DHS wealth distribution is calculated separately for urban and rural areas.*

**Comment 11: Conclusions: this section is rather limited and needs more substance. It reiterates known messages and lacks depth. The limitations need to be thought through more as well as the implications.**

*Authors’ response: Thanks for pointing this out. We have addressed this issue in the revised version of the paper (pp. 29).*
