# Supplementary material for: Exposure to conflicts and the continuum of maternal healthcare: Analyses of pooled cross-sectional data for 452,192 women across 49 countries and 82 surveys
Source: PLoS Med. 2021 Sep 28;18(9):e1003690. doi: 10.1371/journal.pmed.1003690 (PMC8478181; doi:10.1371/journal.pmed.1003690)
Supplement: Attachment — Submitted filename: Point by Point Response to Editors__27.05.21_AR.docx [file pmed.1003690.s005.docx]

**Point-by-point response to Editor and Reviewers’ comments**

Dear Editor

We are grateful to you and the reviewers for their comments and suggestions. The paper has greatly improved from the incorporation of these suggestions. Below we describe the manner in which we have addressed each of the suggestions made by the Editor and Reviewers. Our responses appear in italics.

**Response to Editor’s comments**

Comment 1: To your data statement, please add details of how UCPD data can be obtained.

*Authors’ response: The required information has been added in the revised version-3.*

Comment 2: We ask you to adapt the title slightly to "Exposure to conflicts and the continuum of maternal health care: analysis of pooled cross-sectional data for 452,192 women across 49 countries and 82 surveys".

*Authors’ response: The suggested changes have been made.*

Comment 3: Please use tenses consistently in your abstract, e.g., "We observed ...".

*Authors’ response: The suggested changes have been incorporated.*

Comment 4: Prior to "We observed ..." in your abstract, we suggest adding a sentence to convey some summary statistics, e.g., the proportion of the sample exposed to violent conflict and/or the proportions accessing at least one antenatal service (with 95% CI) in conflict and non-conflict situations.

*Authors’ response: WE have now amended the paper accordingly.*

Comment 5: Where you quote an OR in the abstract, please include 95% CI and p values (e.g., "0.86, 95% CI 0.82-0.91, p<0.001").

*Authors’ response: The suggestion is well-taken and suggested changes have been made.*

Comment 6: Please adapt the abstract so that the final, single sentence of the "Methods and findings" subsection begins "Study limitations include ..." or similar and quotes 2-3 of the study's main limitations. The comment about "The UCDP used ... is generally considered to be reliable." should be removed or relocated to another part of the ms.

*Authors’ response: The suggested changes have been incorporated.*

Comment 7: Late in the abstract, please make that "... statistically significantly and negatively associated ...".

*Authors’ response: The suggested changes have been made.*

Comment 8: Please state explicitly in the Methods section that the study did not have a prespecified analysis plan, assuming this is the case.

Comment 9: The first paragraph of the discussion should summarize the study's findings: one way to achieve this would be to amalgamate the first two current paragraphs of that section, perhaps, with some trimming.

*Authors’ response: The suggested changes have been made.*

Comment 10: Where claiming a "first", "our study is unique", for example, please add "to our knowledge".

*Authors’ response: This comment has been addressed.*

Comment 11: Please use the style "4 or more ANC visits" in the text, although numbers should be spelt out at the start of sentences.

*Authors’ response: The suggestion is well-taken and suggested changes have been made.*

Comment 12: Please remove "hereafter" - an abbreviation on its own will suffice; italics should not be used for emphasis.

*Authors’ response: We have removed the term ‘hereafter’.*

Comment 13: Please remove spaces from within reference call-outs throughout the ms, e.g., "[15-24,30-37]".

*Authors’ response: We have removed space between the references.*

Comment 14: Please use journal name abbreviations consistently in the reference list, e.g., "Lancet" and "BMJ".

*Authors’ response: The suggestion is well-taken and suggested changes have been made.*

Comment 15: Noting reference 7 and others, please list 6 author names, followed by "et al." where appropriate.

*Authors’ response: The suggestion is well-taken and suggested changes have been made.*

Comment 16: In the labels for figure 4, we suggest adapting the text to "Exposure to violence in at least 1 conflict".

*Authors’ response: The suggestion is well-taken and suggested changes have been made.*

Comment 17: In the figures, we suggest adding "Point estimates with 95% confidence interval are shown." or similar to the legends and removing "95% confidence interval" from the figure files themselves.

*Authors’ response: The suggestion is well-taken and suggested changes have been made.*

Comment 18: Please break the attached STROBE checklist out into a separate supplementary file, labelled "S1_STROBE_Checklist" or similar and referred to as such in the text.

*Authors’ response: The suggestion is well-taken and we have uploaded STROBE checklist separately.*

***** Reviewer #1:**

Comment 1: The authors have addressed my concerns and I now recommend publication.

*Authors’ response: Thank you for acknowledging the changes and recommending publication.*

***** Reviewer #2:**

Comment 1: The revised article "Exposure to conflicts and its influence on continuum of maternal health care: Analyses of 452,192 women across 49 countries and 82 surveys" by Rammohan improves on the original article by making some of the exposition more accessible.

*Authors’ response: Thank you for this comment.*

Comment 2: I should note that the responses to the statistical reviewer are largely excellent. The reviewer brought up a couple of comments that commonly reflect an epidemiological perspective. As a quantitative researcher who often straddles methods from epidemiology and economics, it is easy to see why the reviewer would have the concerns about MLM and about the logistic exposition. At the same time, the authors had implemented everything correctly, and the gap was in the language differences between the fields. The latent variable model notation is standard econometrics (and used to motivate the use of LDV models), but not in epi. The variance-covariance clustering of standard errors is conceptually different from MLM, but in this case the single-level model is appropriate.

*Authors’ response: Thank you for acknowledging the changes that we made.*

Comment 3: As noted before, making the exposition heed closer to the standards of medical literature would improve both the understanding and impact of the analysis. The authors have improved that somewhat, but I would urge them to continue hacking at this margin throughout the process of publishing this article.

*Authors’ response: We have done our best to position our manuscript in the medical literature.*

Comment 1: I otherwise have no further comments.

*Authors’ response: Thank you for acknowledging and agreeing to the changes.*

***** Reviewer #3:**

Comment 1: The authors have definitely taken into account the comments and the paper is much stronger as a result. I would say the only shortcoming at the moment are the conclusions. Currently they are rather general and more of an afterthought. There needs to be more action beyond the international aid. What about emergency services and more practical solutions. A simple addition of a paragraph would do.

*Authors’ response: We have added a paragraph on the practical implication emerging from the study.*
