# Supplementary material for: Exposure to conflicts and the continuum of maternal healthcare: Analyses of pooled cross-sectional data for 452,192 women across 49 countries and 82 surveys
Source: PLoS Med. 2021 Sep 28;18(9):e1003690. doi: 10.1371/journal.pmed.1003690 (PMC8478181; doi:10.1371/journal.pmed.1003690)
Supplement: Attachment — Submitted filename: Point by Point Response to Editors_0906 2021_Revision_5.docx [file pmed.1003690.s006.docx]

**Point-by-point response to Editor Comments**

Dear Editor

We are grateful to you and the editors for their comments and suggestions. The paper has greatly improved from the incorporation of these suggestions. Below we describe the manner in which we have addressed each of the suggestions made by the Editor and Reviewers. Our responses appear in italics.

**Response to Editor’s comments**

Comment 1: Unfortunately, we have detected the following problems with your submission that will need to be corrected before we can proceed:

1. Please provide all main figures in .tif or .eps format in the File Inventory. Please note that our file size limit is 10MB. Please remove the embedded figures from your manuscript.
2. -Currently figure 4 is cited on page 16 of the manuscript but on page 33 only figure 1-3 are listed. In the file Inventory figures are named fig 3 and 4.

***Authors Response: As suggested, we have provided all main figures in .tif or .eps format in the File Inventory. On page 33, now we have indicated list of all four figures.***
